# Analysis of cell signaling profiles induced by DNA aptamer-based FGFR1 agonist

**DOI:** 10.1007/s44211-024-00660-1

**Published:** 2024-09-09

**Authors:** Junya Hoshiyama, Yuri Hayata, Akihiro Eguchi, Jumpei Morimoto, Ryosuke Ueki, Shinsuke Sando

**Affiliations:** 1 Department of Chemistry and Biotechnology, Graduate School of Engineering, The University of Tokyo, 7-3-1 Hongo, Bunkyo-ku, Tokyo, 113-8656 Japan; 2 Department of Bioengineering, Graduate School of Engineering, The University of Tokyo, 7-3-1 Hongo, Bunkyo-ku, Tokyo, 113-8656 Japan

**Keywords:** Artificial agonist, DNA aptamer, Fibroblast growth factor receptor, Phosphoproteomics, Receptor signaling

## Abstract

DNA aptamers have attracted attention as an alternative modality for biomolecules due to their excellent target binding specificity and thermal stability, and they are also expected to be applied as artificial agonists for receptor proteins. DNA aptamer agonist TD0 targeting the receptor of fibroblast growth factor (FGFR), which plays an important role in the fields of wound healing and regenerative medicine, has been reported to induce cellular responses as well as its native ligands. However, it was also noted that there were some different responses upon long-term stimulation, suggesting that the intracellular signals induced by DNA aptamer agonist TD0 are different from those of natural ligands. In this paper, we comprehensively analyzed the intracellular signals induced by DNA aptamer agonist TD0 targeting FGFR1, and compared them with those by natural protein ligand FGF2. It was found that the intracellular signals were highly similar for short-term stimulation. On the other hand, the receptor and the downstream cellular signals showed different activation behaviors for long-time stimulation. Evaluating the stability and sustained activity of DNA aptamer agonist TD0 and FGF2 in the medium suggested that ligand stability may be important in properly regulating cellular responses.

**Graphical Abstract:**

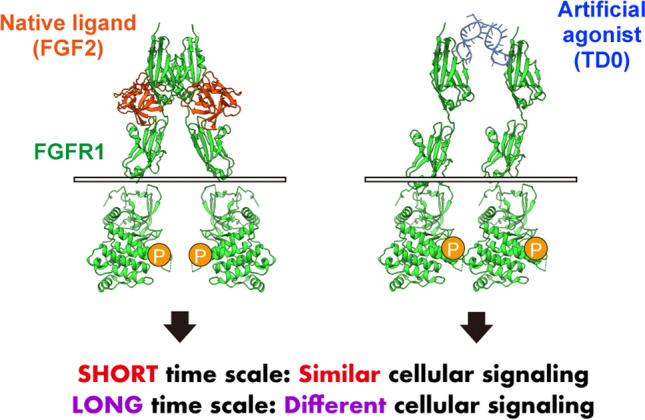

## Introduction

**Fig. 1:**
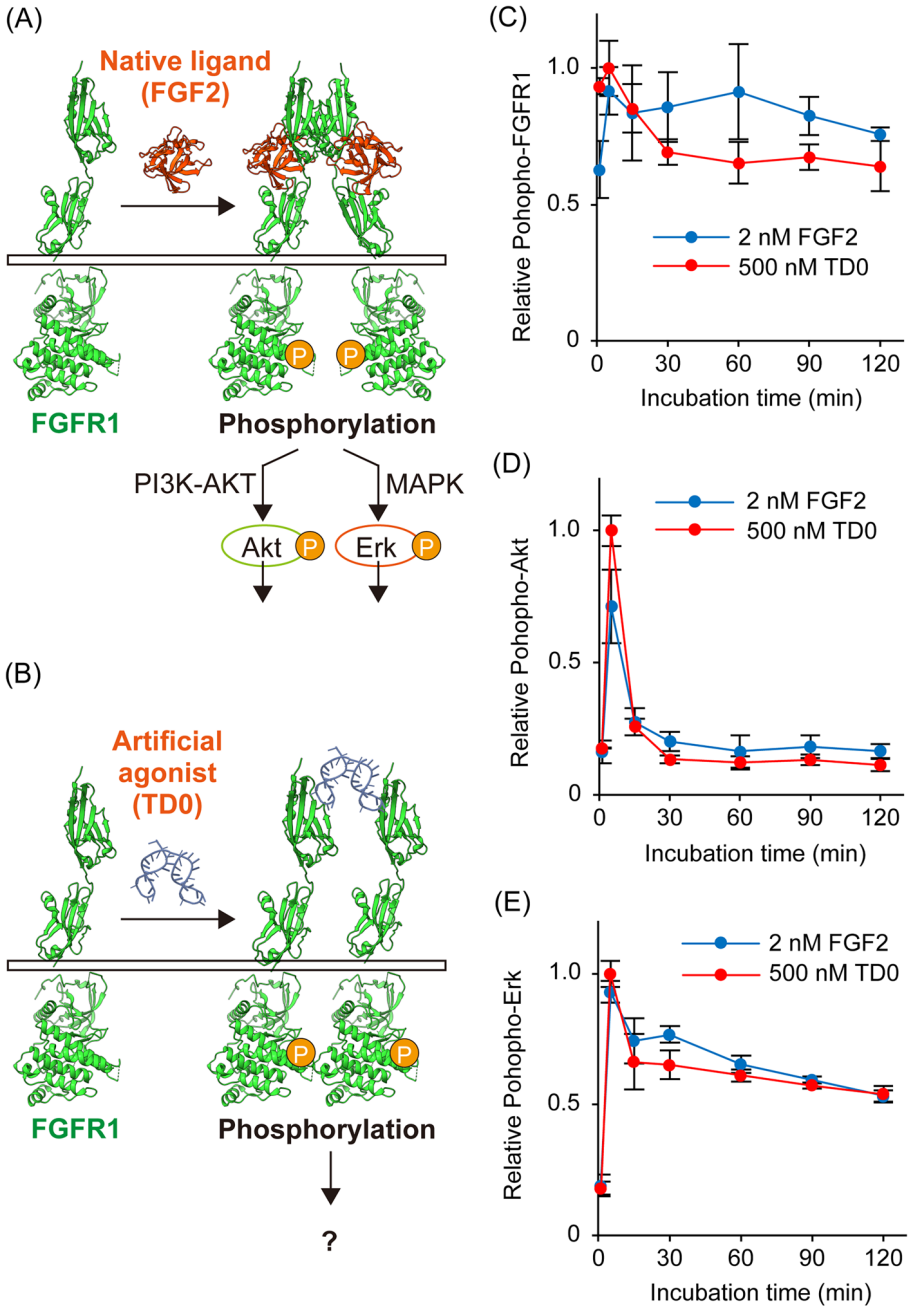
**A**, **B** Schematic diagram of FGFR1 and its downstream signaling pathways activated by (A) native ligand (FGF2) or (B) DNA-based artificial agonist (TD0). **C**, **D**, **E** Relative phosphorylation levels of (**C**) FGFR1, (**D**) Akt, or (**E**) Erk induced by FGF2 (2 nM, blue circle) or TD0 (500 nM, red circle) for 1, 5, 15, 30, 60, 90, and 120 min. Data are normalized relative to the value of the sample stimulated by 500 nM TD0 for 5 min. Error bars indicate S.D. (*N* = 3)

Fibroblast growth factor receptors (FGFRs) activate downstream intracellular signaling by the binding of their native ligands, fibroblast growth factors (FGFs), and regulate various functions such as cell growth, differentiation, and migration (Fig. 1A) [1–4]. Due to their essential activity, FGFs have been applied in the fields of wound healing, stem cell culture, and induction of differentiation [5–8]. Despite their importance, FGFs have problems such as high production cost, low thermal stability, and heterogeneous activity due to the production from the biological hosts. Therefore, the development of artificial agonists that can serve as alternatives has been demanded.

DNA aptamers are nucleic acid sequences with specific binding ability to target biomolecules. They are promising as a modality for artificial agonists because of their reversible thermal stability of higher-order structures and the ease of ensuring uniformity through chemical synthesis [9]. DNA aptamers with binding ability to membrane receptors have been obtained and applied to the design of artificial agonists [10–14]. For FGFRs, we have reported the artificial agonist TD0, designed by dimerizing FGFR1-binding DNA aptamer, to induce the phosphorylation of FGFR1 and maintain the undifferentiated state of iPS cells as with FGF2 (Fig. 1A and B) [14].

However, it was also reported that TD0 could not maintain the undifferentiated state of iPS cells in long-term cell culture [14], suggesting that there were some differences between the intracellular signals induced by natural protein-based ligands and DNA aptamer-based artificial agonists. Although various agonists based on DNA aptamers have been developed, the cell signaling patterns induced by DNA aptamer agonists have not been well investigated, including the differences in TD0 *vs* FGF2. In this research, to further understand the receptor signaling mechanism and establish strategies for designing high-performance artificial agonists, we conducted a comprehensive analysis of intracellular signaling induced by TD0 or FGF2 and evaluated the similarities and differences in these ligand activities.

## Experimental

### Oligonucleotides

Oligonucleotide (TD0: 5’ GCC GCG TCT TTA TGG CTG GGG ATG GTG TGG GTT GCG GCG CCG CGT CTT TAT GGC TGG GGA TGG TGT GGG TTG CGG C 3’) [14] was purchased from Eurofins Genomics. Before usage, the oligonucleotide was folded by heating at 95 °C for 5 min, followed by cooling at 0.1 °C/sec to 25 °C in DPBS using a thermal cycler.

### Cell culture and preparation of cell lysates

A204 cells (ATCC) were cultured in DMEM (#08456–65, Nacalai Tesque) supplemented with 10% fetal bovine serum (FBS, COSMO BIO) and 1% Antibiotic–Antimycotic (#09366–44, Nacalai Tesque) at 37 °C in 5% CO_2_ atmosphere.

A204 cells were seeded in 35 mm dishes and cultured in DMEM supplemented with 10% FBS and 1% Antibiotic–Antimycotic. After overnight incubation, the medium was replaced with a starvation medium (DMEM supplemented with 1% Antibiotic–Antimycotic) and cultured for 24 h. After the starvation, the medium was replaced with the fresh starvation medium, and the cells were incubated in the presence or absence of recombinant FGF2 (2 nM, PeproTech, #100-18B) or TD0 (500 nM) for the indicated time at 37 °C. The cells were washed twice with DPBS and lysed with a lysis buffer (20 mM Tris–HCl pH 8.0, 150 mM NaCl, 1% TritonX-100, 1 mM EDTA, 2.5 mM sodium pyrophosphate, 1 mM beta-glycerophosphate, 1 μg/mL leupeptin, and 10% glycerol) supplemented with 1 mM 4-(2-aminoethyl)benzolsulfonylfluoride hydrochloride (AEBSF). The lysates were incubated on ice for over 30 min, followed by centrifugation at 10,000 × g for 15 min at 4 °C. The supernatants were recovered and used for capillary electrophoresis immunoassay.

### Capillary electrophoresis immunoassay

Jess^™^ Simple Western system (ProteinSimple) was used for the quantification of proteins, following the manufacturer’s protocol for the separation module (#SM-W004), anti-rabbit detection module (#DM-001), and total protein detection module (#DM-TP01). Cell lysates were mixed with 10 × Sample buffer and Fluorescent 5 × Master mix (ProteinSimple), then incubated at 95 °C for 5 min. Antibodies were prepared in antibody diluent by the indicated ratio: anti-phospho-FGFR1 Tyr653/654 (#52928S, Cell Signaling Technology, 1:100), anti-phospho-ERK Thr202/Tyr204 (#4370S, Cell Signaling Technology, 1:250), and anti-phospho-AKT Ser473 (#4060S, Cell Signaling Technology, 1:100). Compass Simple Western software (ProteinSimple) was used to quantify chemiluminescence. The peaks were fitted by the Gaussian method. Corrected peak areas, that were normalized by total protein detected, were used for analysis. Student’s t-test was performed to indicate statistical significance.

### SILAC-based phosphoproteomics

A204 cells were grown in DMEM for SILAC (#88,364, Thermo Fisher Scientific) supplemented with 10% dialyzed FBS (#A3382001, Thermo Fisher Scientific), 1% Antibiotic–Antimycotic, and either “light” or “heavy” amino acids. The “light” SILAC media were supplemented with L-lysine and L-arginine (#L8662-25G and #A6969-25G, Thermo Fisher Scientific), and “heavy” SILAC media were supplemented with L-lysine-U-^13^C_6_-^15^N_2_ and L-arginine-U-^13^C_6_-^15^N_4_ (#123-06081 and #010-24041, Fujifilm Wako Pure Chemical Corporation). The cells were cultured in these SILAC media for 1 week before the analysis. The “heavy” labeled cells were treated with FGF2 (2 nM) or TD0 (500 nM) for 5 min at 37 °C in the “heavy” SILAC media, and the “light” labeled cells were treated with vehicle (DPBS) in the “light” SILAC media. The cells were washed twice with DPBS and proteins were extracted with TRIZOL (#15,596,026, Thermo Fisher Scientific), followed by isopropanol precipitation and resuspension in protein solubilization buffer (50 mM bicine pH 8.5 and 8 M guanidine-HCl). The samples were centrifuged at 10,000 × g for 10 min, and the supernatants were collected. Equal amounts of protein from “heavy” and “light” samples (0.15 mg) were mixed and phosphatase inhibitors (sodium orthovanadate, β-glycerophosphate, and sodium pyrophosphate) were added. Proteins were reduced with 25 mM tris(2-carboxyethyl)phosphine at 37 °C for 15 min and free cysteine residues were alkylated with 25 mM iodoacetamide at 37 °C for 30 min in the dark with gentle shaking. Proteins were digested with Lys-C (Fujifilm Wako Pure Chemical Corporation) at an enzyme-protein ratio of 1:100 at 37 °C for 3 h, diluted in 100 mM Tris–HCl (pH 8.5) containing 2 M urea, and subsequently digested with trypsin (Promega) at an enzyme-protein ratio of 1:100 at 37 °C overnight. The addition of trifluoroacetic acid (TFA) inactivated the trypsin. Digested peptides were desalted with MonoSpin C18 spin column (GL Science), evaporated in vacuo, and reconstituted in 200 µL of 80% acetonitrile with 0.1% TFA. Phosphopeptides in samples were enriched using Fe(III)-IMAC cartridges (5 μL) on an AssayMAP Bravo platform (Agilent) at the flow rate of 5 μL/min according to the manufacturer’s protocol. Phosphopeptides were eluted three times with different conditions; 20 µL of 20% acetonitrile with 1% TFA, 25% acetonitrile with 5% ammonia, and 25% acetonitrile with 5% pyrrolidine, respectively. 2nd and 3rd eluates were mixed and acidified with TFA. 1st and 2nd/3rd eluates were concentrated and desalted using GL-Tip SDB (GL Science) and each eluate was measured twice by Q-Exactive (Thermo Fisher Scientific) coupled with a system of capillary reverse-phase liquid chromatography. All MS raw files were processed in Proteome Discoverer 2.4.1.15 (Thermo Fisher Scientific), and peptide identification was performed using SEQUEST (Thermo Fisher Scientific) against peptide sequences of Homo Sapiens (SwissProt TaxID: 9606), using SILAC 2plex (Arg10, Lys8) as a quantification method. Phosphopeptides were filtered based on site probability (≥ 75%) and phosphopeptides quantified in all conditions and duplications were selected for the following bioinformatics analysis.

## Results and discussion

### TD0 and FGF2 exhibit similar FGFR1 activation in short-term stimulation

We initially investigated the time dependency of FGFR1 phosphorylation levels upon stimulation. A previous report demonstrated that stimulation with 500 nM TD0 induces FGFR1 phosphorylation in A204 cells with an intensity comparable to 2 nM FGF2 [14]. Therefore, A204 cells were stimulated with 2 nM FGF2 or 500 nM TD0 for 1, 5, 15, 30, 60, 90, and 120 min. Subsequently, the phosphorylation of FGFR1 in the lysate was quantified using capillary electrophoresis immunoassay (CEI). The relative phosphorylation levels from the value of the sample stimulated by 500 nM TD0 for 5 min are shown with error bars (S.D. of N = 3) at each time point (Fig. 1C). The results showed that FGFR1 phosphorylation reached maximal 5 min after stimulation for FGF2 and TD0. After 5 min, phosphorylation levels decreased slightly in TD0, but both ligands maintained sustained phosphorylation throughout the observed period.

Additionally, to gain insight into intracellular signaling, the phosphorylation levels of Akt and Erk, representative downstream signaling proteins of FGFR1, were also quantified (Fig. 1D and E). Activation of FGFR and MAPK pathway, to which Erk belongs, is suggested to be important for the maintenance of the undifferentiated state of stem cells [15]. Both Akt and Erk showed maximal phosphorylation levels at 5 min, similar to the phosphorylation profile of FGFR1. The phosphorylation level of Akt decreased rapidly, whereas Erk showed a relatively sustained activation. These trends were observed in both FGF2 and TD0, indicating that FGF2 and TD0 similarly activate downstream signals up to 120 min after stimulation.

### TD0 and FGF2 induce similar patterns of intercellular signaling in short-term stimulation

**Fig. 2:**
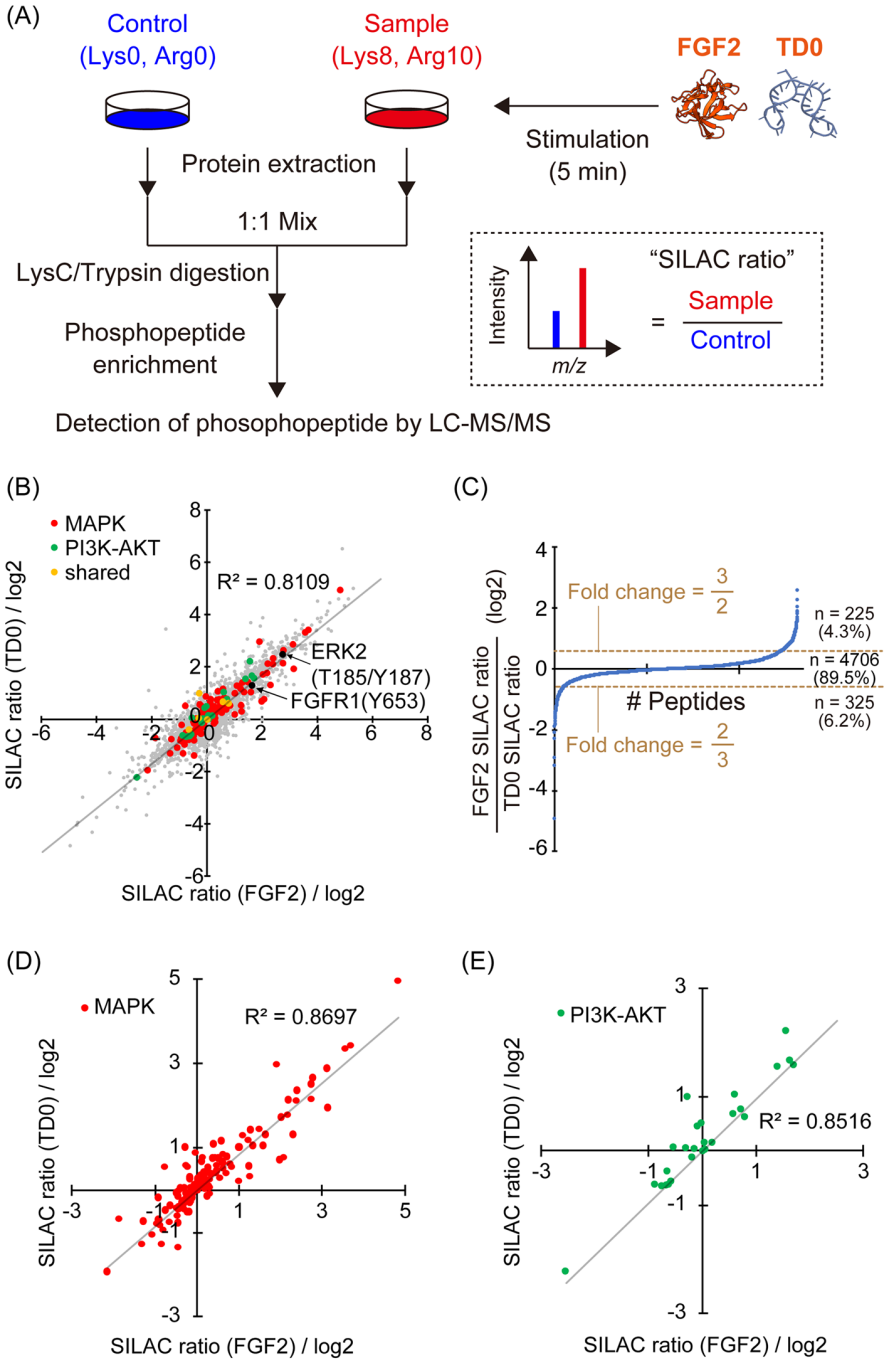
**A** Overview of the experimental protocol of SILAC-based phosphoproteomics. **B** Scatter plot of identified phosphopeptides according to the log2-scaled SILAC ratio of FGF2 or TD0. Peptides belonging to both MAPK and PI3K-AKT pathways are indicated as “shared”. **C** Peptide ranking according to the log2-scaled SILAC ratio between FGF2 and TD0. **D**, **E** Scatter plots of phosphopeptides belonging to the **D** MAPK pathway or **E** PI3K-AKT pathway

We performed a comprehensive analysis of intracellular signaling by stable isotope labeling by amino acid in cell culture (SILAC)-based quantitative phosphoproteomics using the cell lysates of A204 cells treated with FGF2 or TD0 for 5 min (Fig. 2A). Proteins extracted from the cells were mixed to those from unlabeled cells with no stimulation in a 1:1 ratio, followed by LysC/trypsin digestion and phosphopeptide enrichment using Fe(III)-immobilized metal ion affinity chromatography (IMAC) beads. Samples were analyzed by liquid chromatography-tandem mass spectrometry (LC-MS/MS) consisting of the system of capillary reverse-phase HPLC and Q-Exactive mass spectrometer. The raw data obtained were analyzed by Proteome Discoverer (Thermofisher Scientific), and 5,256 phosphopeptides were selected for further analysis based on the following criteria: (1) detection in all duplicate samples under all conditions and (2) identification of phosphorylation sites with a site probability of 75% or higher. The ratio of the amount of each phosphopeptide between stimulated samples and non-stimulated samples (SILAC ratio) was calculated and plotted in the scatter plot (Fig. 2B). In this plot, peptides belonging to the PI3K-Akt and MAPK pathways, which are the representative downstream signaling pathways of FGFR1 shown in Fig. 1A, were highlighted in green and red, respectively (Fig. 2B). The correlation coefficient (R^2^) calculated from all peptides was 0.8109, suggesting similar patterns of intracellular signaling induced by TD0 and FGF2. In addition, the ratio of the FGF2 SILAC ratio and TD0 SILAC ratio for each peptide was plotted (Fig. 2C), indicating that a high percentage of peptides have a similar degree of change in phosphorylation by FGF2 and TD0 (2/3 < Fold Change < 3/2, 89.5%). Furthermore, focusing on the phosphorylated peptides belonging to PI3K-Akt and MAPK pathways, the R^2^ values were 0.8516 and 0.8697, respectively (Fig. 2D and E). Since these pathways are key downstream signaling of FGFR1, the results suggest the higher similarity of cellular signal induction between FGF2 and TD0 was caused by FGFR1 activation.

### TD0 and FGF2 exhibit distinct FGFR1 activation in long-term stimulation

**Fig. 3:**
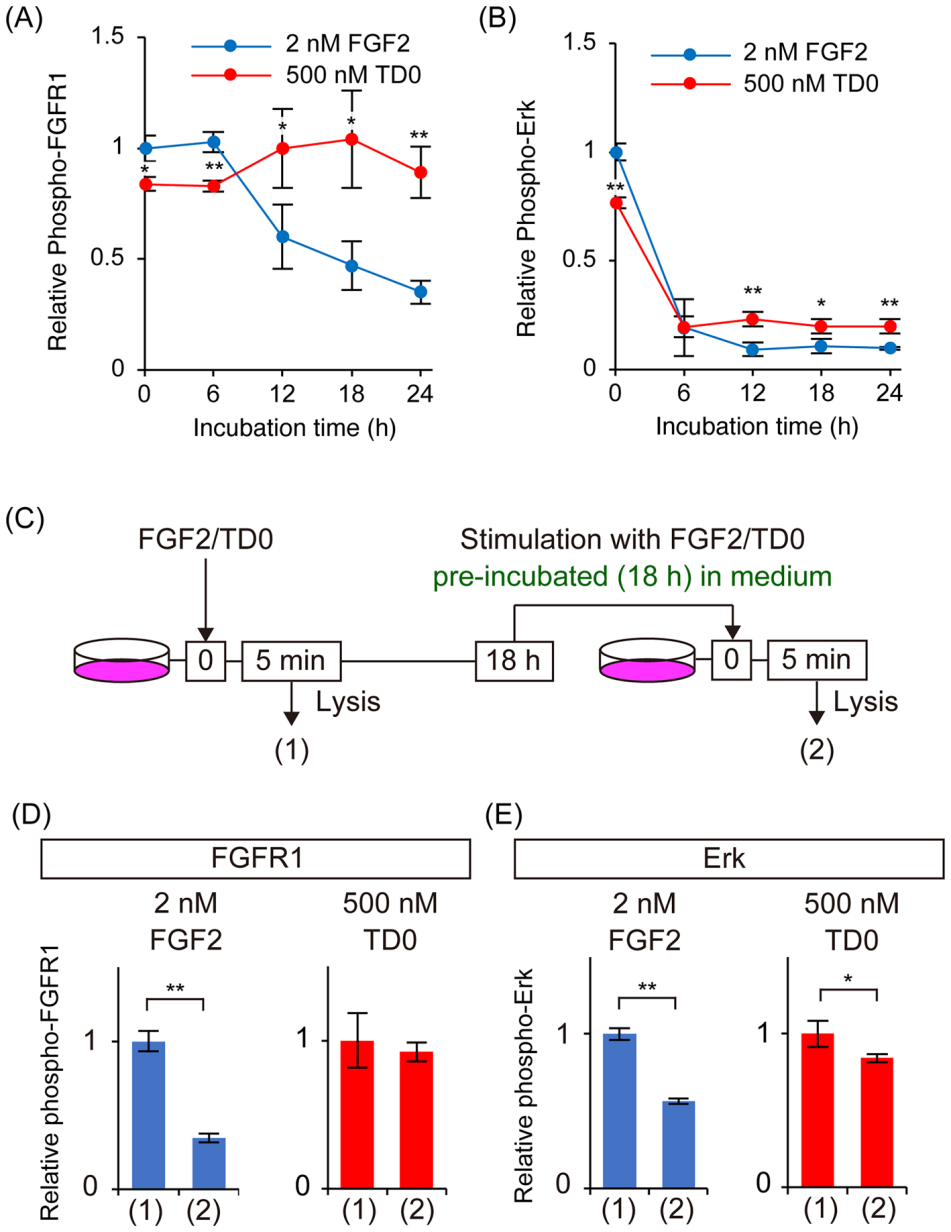
**A**, **B** Phosphorylation levels of (**A**) FGFR1 or (**B**) Erk induced by FGF2 (2 nM, blue circle) or TD0 (500 nM, red circle) for 5 min, 6, 12, 18, and 24 h. Data are normalized relative to the value of the sample stimulated by 2 nM FGF2 for 5 min. Error bars indicate S.D. (*N* = 3). *p < 0.05, **p < 0.01 v.s. FGF2 (**C**) Overview of the experimental protocol for evaluating ligand stability. **D**, **E** Phosphorylation levels of (**D**) FGFR1 or (**E**) Erk induced by FGF2 (2 nM, blue bar) or TD0 (500 nM, red bar) in lysates (1) and (2) in Fig. 3C. Data are normalized against the values for samples stimulated by 2 nM FGF2 or 500 nM TD0 for 5 min (lysate (1)). Error bars indicate S.D. (*N* = 3). *p < 0.05, **p < 0.01

The results of phosphoproteomics with cell lysates at 5 min post-stimulation suggested that TD0 could induce intracellular signaling similar to FGF2 for a relatively short duration. However, given that differences in cellular responses were observed in long-term stem cell cultures in the previous report, we next evaluated the phosphorylation patterns of FGFR1 and Erk on a longer time scale. A204 cells were treated with 2 nM FGF2 or 500 nM TD0 for 5 min, 6, 12, 18, and 24 h, and phosphorylated FGFR1 and Erk in the cell lysate were quantified by CEI. The relative phosphorylation levels from the value of the sample stimulated by 2 nM FGF for 5 min are shown with error bars (S.D. of N = 3) at each time point (Fig. 3A and B). The results showed that FGF2 indued higher FGFR1 phosphorylation levels up to 6 h after stimulation, while TD0 showed higher and more sustained FGFR1 phosphorylation levels beyond 12 h. In addition, Erk showed similar or slightly higher phosphorylation levels by TD0 than FGF2 after 12 h. Consequently, TD0 and FGF2 induced distinct phosphorylation patterns upon prolonged stimulation, which may be one of the reasons for the differences in cell behavior in long-term cell culturing reported previously [14].

### TD0 and FGF2 have different stability in the medium

To determine which properties of the ligands are responsible for these different phosphorylation patterns in long-term stimulation, we conducted further investigations focusing on time-dependent changes in the activity of each ligand in the culture medium. Cells stimulated with 2 nM FGF2 or 500 nM TD0 for 5 min showed activation of FGFR1 and Erk (Fig. 3C-E, lysate (1)). In contrast, when newly prepared cells were stimulated for 5 min in media containing 2 nM FGF2 or 500 nM TD0, which were pre-incubated with cells for 18 h, activation of FGFR1 and Erk was observed in medium containing TD0 but was suppressed in medium containing FGF2 (Fig. 3C-E, lysate (2)). This result suggests that, in contrast to FGF2, TD0 remains active even after 18 h of incubation.

Previous reports indicated that the activity of FGF2 in the medium without heparin attenuates over time during cell culture due to thermal denaturation, aggregation, and enzymatic degradation [16]. In contrast, TD0 is considered to be relatively stable due to its unique DNA G-quadruplex structure. Furthermore, TD0 is added to the culture medium at high concentrations (500 nM) to achieve FGFR1 activation comparable to that by FGF2, suggesting that large amounts of TD0 may remain active after long-term culture. Therefore, the differences in the duration of signaling input to FGFR1 might contribute to the different cellular responses in long-time cell cultures between FGF2 and TD0.

## Conclusion

This study characterized intracellular signaling induced by the DNA aptamer-based artificial FGFR1 agonist TD0. We compared the pattern of signal induction with that of the native ligand FGF2, aiming to understand the differences in cellular responses observed in long-term cell culture experiments.

Our findings revealed that, in a shorter timescale, the artificial agonist TD0 induces cellular signaling that is highly similar to that induced by FGF2. FGF2 is thought to form a 2:2 complex with FGFR1 to induce FGFR1 activation [2], while TD0 dimerizes FGFR1 to form a 1:2 complex with FGFR1 and induces FGFR1 proximity and activation (Fig. 1A and B). Although this bivalent aptamer strategy has been widely used as a design guideline for aptamer agonists [11, 12, 17, 18], the ability of such agonists to induce intracellular signals akin to those of natural ligands has yet to be confirmed. The observed similarity in cellular signaling, between TD0 and FGF2, underscores the efficacy of the receptor dimerization strategy by bivalent aptamers in achieving comparable levels of intracellular signaling. Further phosphoproteomics investigation using A204 cells with long-time stimulation and a similar study using iPS cells would enable a deeper discussion.

On the other hand, differences in the phosphorylation profiles induced by TD0 and FGF2 became apparent in cell cultures lasting longer than 6 h. TD0 was able to sustain cell activation for a longer duration compared to FGF2. This could be attributed to the stability of TD0 or the fact that TD0 requires a 100-fold higher initial concentration than FGF2 [14], possibly because of the differences in the affinity to FGFR or the efficacy of FGFR dimerization with proper orientation. This observation highlights the importance of developing aptamers that degrade gradually or can act at lower initial concentrations for future agonist designs intended to mimic the activity of FGF2 more closely. In addition, the fact that TD0 exhibits very high FGFR1 selectivity without binding to other members of the FGFR family [19], may also play a role in this difference. A further detailed investigation into these aspects is essential to understand the nuances and differences in intracellular signaling induced by artificial agonists compared to natural agonistic ligands.

## Data Availability

The data that support the findings of this study are available upon reasonable request.
